# Residual and bidirectional LSTM for epileptic seizure detection

**DOI:** 10.3389/fncom.2024.1415967

**Published:** 2024-06-17

**Authors:** Wei Zhao, Wen-Feng Wang, Lalit Mohan Patnaik, Bao-Can Zhang, Su-Jun Weng, Shi-Xiao Xiao, De-Zhi Wei, Hai-Feng Zhou

**Affiliations:** ^1^Chengyi College, Jimei University, Xiamen, China; ^2^Shanghai Institute of Technology, Shanghai, China; ^3^London Institute of Technology, International Academy of Visual Arts and Engineering, London, United Kingdom; ^4^National Institute of Advanced Studies, Bangalore, India; ^5^Marine Engineering Institute, Jimei University, Xiamen, China

**Keywords:** EEG, epilepsy, epileptic seizure detection, ResNet, LSTM, deep learning

## Abstract

Electroencephalogram (EEG) plays a pivotal role in the detection and analysis of epileptic seizures, which affects over 70 million people in the world. Nonetheless, the visual interpretation of EEG signals for epilepsy detection is laborious and time-consuming. To tackle this open challenge, we introduce a straightforward yet efficient hybrid deep learning approach, named ResBiLSTM, for detecting epileptic seizures using EEG signals. Firstly, a one-dimensional residual neural network (ResNet) is tailored to adeptly extract the local spatial features of EEG signals. Subsequently, the acquired features are input into a bidirectional long short-term memory (BiLSTM) layer to model temporal dependencies. These output features are further processed through two fully connected layers to achieve the final epileptic seizure detection. The performance of ResBiLSTM is assessed on the epileptic seizure datasets provided by the University of Bonn and Temple University Hospital (TUH). The ResBiLSTM model achieves epileptic seizure detection accuracy rates of 98.88–100% in binary and ternary classifications on the Bonn dataset. Experimental outcomes for seizure recognition across seven epilepsy seizure types on the TUH seizure corpus (TUSZ) dataset indicate that the ResBiLSTM model attains a classification accuracy of 95.03% and a weighted F1 score of 95.03% with 10-fold cross-validation. These findings illustrate that ResBiLSTM outperforms several recent deep learning state-of-the-art approaches.

## 1 Introduction

Epilepsy is a neurological disorder stemming from sudden irregular nerve cell discharges in the brain (Fisher et al., 2017). It impacts a global population of over 70 million across all age groups (Thijs et al., 2019). The timely and precise identification of epileptic conditions, coupled with appropriate treatment, holds the potential to alleviate patient suffering. Electroencephalogram (EEG) play an important role in detecting epilepsy, as it captures rich physiological and pathological information, reflecting brain nerve cells electrophysiological activities on the scalp surface or cerebral cortex (Foreman and Hirsch, 2012; Devinsky et al., 2018). Traditional diagnosis and treatment of epilepsy require neurologists to manually sift through extensive EEG recordings, a process often hindered by its labor-intensive, time-consuming, and subjective nature, especially in identifying brief, low-amplitude events (Cendes et al., 1997; Sheth et al., 2014; Kramer et al., 2019). Consequently, there is an urgent need for an automated and reliable detection approach. Such a method would not only alleviate the burden on neurologists but also enable prompt and effective treatment for patients (Cho and Jang, 2020). In response to this need, a considerable volume of research has been dedicated to enhancing epileptic seizure detection.

The traditional predominant approach in epilepsy detection combines conventional signal processing with machine learning techniques (Subasi and Gursoy, 2010). These methodologies primarily revolve around feature extraction and classification. In feature extraction, researchers manually construct EEG signal features based on empirical knowledge and observations. A wide array of features has been derived from the time domain (Gotman, 1982), frequency domain (Qu and Gotman, 1997), and time-frequency domain (Saab and Gotman, 2005) for seizure EEG recognition. Additionally, various nonlinear features, including the Hurst exponent (Lahmiri, 2018), entropies (Acharya et al., 2012), Lyapunov exponent (Elger et al., 2005), and fractal dimension (Zhang et al., 2015), have been utilized for epilepsy seizure detection. Various classifiers, such as logistic regression (Claassen et al., 2004), Bayesian (Saab and Gotman, 2005), k-nearest neighbor (KNN) (Acharya et al., 2012), random forest (RF) (Mursalin et al., 2017), support vector machines (SVM) (Park et al., 2011), and artificial neural networks (ANN) (Ramgopal et al., 2014), have subsequently been applied for identifying seizure activities. While algorithms based on hand-crafted feature engineering have achieved significant success in detecting epileptic seizures (Sharma and Pachori, 2015; Sharma et al., 2017), distinguishing between epileptic and non-epileptic EEG signals remains a challenge due to data noise, artifacts, and the variability in seizure morphology (Tao et al., 2017).

The emergence of deep learning (DL) (LeCun et al., 2015) techniques in various domains such as computer vision (Chen et al., 2021), natural language processing (Young et al., 2018), and speech recognition (Khalil et al., 2019) has demonstrated substantial performance improvements. It employs ANN with multiple layers to extract intricate features from data. Its primary objective is to capture high-level abstractions within data by employing intricate architectures comprising multiple non-linear transformations. Therefore, DL techniques can automatically extract intrinsic features from EEG signals, and achieve end-to-end epileptic seizure detection (Zhang et al., 2020; Zhao et al., 2020; Zhao and Wang, 2020). Hence, this paper investigates DL-based epileptic seizure detection approaches. These models are categorized as convolutional neural network (CNN)-based models, recurrent neural network (RNN)-based models, and hybrid models.

CNNs can efficiently extract local spatial features from raw EEG data without the need for hand-crafted feature extractors. Johansen et al. (2016) developed a CNN featuring filters of various sizes at the input layer, leaky ReLUs as activation functions, and a sigmoid output layer, aimed at automated detection of spikes in EEG recordings of epileptic patients. Their model achieved an AUC of 0.947 on scalp EEG recordings from five patients with diagnosed epilepsy. Tang et al. (2021) employed a CNN comprising two convolutional layers for classifying raw time-series EEG data. Their approach achieved an AUC of 0.752 in a dataset encompassing 930 seizures across nine distinct seizure types. Acharya et al. (2018) developed a 13-layer deep CNN algorithm to discern healthy, interictal, and ictal EEG recordings, achieving accuracy of 88.67%. Ullah et al. (2018) introduced an ensemble of pyramidal one-dimensional CNN (P-1D-CNN) models for binary and ternary epilepsy detection, featuring 61% fewer parameters compared to standard CNN models, thereby enhancing generalizability. Türk and Özerdem (2019) transformed EEG records into two-dimensional frequency-time scalograms using continuous wavelet transform and utilized a CNN to analyze these scalogram images. Roy et al. (2020) performed a thorough search space exploration to evaluate the efficacy of a variety of preprocessing approaches, machine learning algorithms, and hyperparameters to classify seven seizure types using the TUSZ dataset. Their research in the development of a CNN model achieved a weighted F1 score of 0.722. Raghu et al. (2019) introduced a CNN-based framework that transforms EEG time series into spectrogram stacks, tailored for CNN input. This approach was applied to an eight-class classification challenge using the TUSZ dataset. The accuracy was meticulously evaluated through four distinct CNN models: AlexNet (84.06%), VGG16 (79.71%), VGG19 (76.81%), and the basic CNN model (82.14%). Shankar et al. (2021) utilized the Gramian Angular Summation Field (GASF) to convert 1D EEG signals into 2D images, followed by employing a CNN for automatic feature extraction and classification. This approach yielded impressive accuracies in seizure type classification, reaching up to 96.01% for binary, and 89.91, 84.19, and 84.20% for multiclass categories – 3, 4, and 5 respectively, on the TUSZ dataset.

RNN-based methodologies are recognized as effective DL solutions for time-series problems, finding widespread application in diverse domains. Long short-term memory (LSTM), gated recurrent unit (GRU), bidirectional long short-term memory (BiLSTM) and bidirectional gated recurrent unit (BiGRU) represent variant models derived from the traditional RNN architecture. Güler et al. (2005) formulated an Elman RNN model integrated with Lyapunov exponents to distinguish healthy, interictal, and ictal EEG signals. Hussein et al. (2018) proposed an optimized deep neural network architecture featuring a single-layer LSTM for robust epileptic seizure detection using EEG signals, even in noisy and real-life conditions. Tuncer and Bolat (2022b) adopted instantaneous frequency and spectral entropy to extract features from raw EEG signals, employing BiLSTM as the classifier. Their model achieved 99% accuracy in binary classification on the Bonn epilepsy dataset. Zhang et al. (2022) conducted wavelet transforms as a preprocessing step for EEG signals, subsequently employing a BiGRU network, along with a series of post-processing steps to generate discriminant results regarding seizure presence. Their model achieves an average sensitivity of 93.89% and an average specificity of 98.49% on the CHB-MIT scalp EEG database. Tuncer and Bolat (2022a) integrated classifiers such as KNN, RF, SVM, and LSTM to achieve an accuracy of 95.92% for four-category seizure type detection and 98.08% for two-category seizure type classification within the TUSZ scalp EEG dataset.

To leverage the advantages of both CNNs and RNNs, hybrid models combining these architectures have been proposed. These hybrid models are capable of capturing both the spatial features and the temporal dynamics of EEG signals. Typically designed in series or parallel, hybrid DL models combine CNN and LSTM. Xu et al. (2020) proposed a serial connection between CNN and LSTM, where CNN first extracts spatial features from normalized EEG sequences, subsequently utilized by LSTM to learn temporal dependencies. Their method achieved recognition accuracies of 99.39 and 82.00% for two-category and five-category epileptic seizure detection tasks, respectively, on the Bonn university epilepsy dataset. Furthermore, several analogous studies have utilized varying numbers of convolutional layers or LSTM layers (Yang et al., 2021; Pandey et al., 2023; Shanmugam and Dharmar, 2023; Wang et al., 2023; Yu et al., 2023). Similarly, Ahmad et al. (2023) combined CNN and BiLSTM in a series, implementing truncated backpropagation through time to efficiently capture spatial and temporal sequence information while minimizing computational complexity. Their model achieved accuracies of 99.41 and 84.10% for two-category and five-category classifications, respectively, on the Bonn epilepsy dataset. Affes et al. (2019) introduced a convolutional gated recurrent neural network (CGRNN) for predicting epileptic seizures from EEG data, capturing both temporal and frequency aspects of the signals. The CGRNN, tested on data from the Children’s Hospital of Boston, achieves an average sensitivity of 89%, a mean accuracy of 75.6%, and a false positive rate of 1.6 per hour, with performance varying across patients. Ma et al. (2023) introduced a parallel multi-channel feature fusion model, CNN-Bi-LSTM, featuring dot-product attention for weighted electrode channel output classification. Their model achieved accuracies of 94.83 and 77.62% for three-category and five-category classifications, respectively. Qiu et al. (2023) introduced the DARLNet model, a parallel combination of the ResNet and LSTM network. They further enhanced recognition accuracy by introducing a difference layer and channel attention mechanism. Their model achieved a recognition accuracy of 90.17% for the five-category classification task.

Moreover, the recognized modesty of the aforementioned hybrid models persists in multi-classification seizure detection. Consequently, we propose a novel hybrid network model based on residual and BiLSTM (ResBiLSTM) to enhance recognition accuracy across various classification challenges. The results reveal the superior performance of the proposed ResBiLSTM model compared to several existing hybrid methods.

The rest of this paper is organized as follows. Section 2 introduces the datasets and the proposed methodology. Section 3 covers the experimental results and analysis. Section 4 discusses the results and compare the performance with other DL algorithms. Finally, the study’s conclusions are presented in Section 5.

## 2 Materials and methods

### 2.1 The benchmark datasets

#### 2.1.1 The Bonn dataset

The Bonn dataset used in this study was acquired by a research team at the University of Bonn and have been extensively used for research on epilepsy seizure detection (Andrzejak et al., 2001). These segments were selected and cut out from continuous multichannel EEG recordings after visual inspection for artifacts. The dataset consists of five sets (denoted A-E) each containing 100 single-channel EEG segments of 23.6-s duration. Each EEG segment was recorded using a standard 10–20 electrode placement system at a sampling rate of 173.61 Hz, so each segment contains 4,097 data points.

Sets A and B consisted of segments taken from scalp EEG recordings that were carried out on five healthy volunteers while they were relaxed in an awake state with eyes opened (A) and eyes closed (B), respectively. Sets C, D, and E were acquired from five patients. Set C was recorded from the hippocampal formation of the opposite hemisphere of the brain. Set D were recorded from within the epileptogenic zone. Sets C and D consists of EEG signals measured during seizure free intervals. Set E only contained seizure activity.

#### 2.1.2 The TUSZ dataset

The TUSZ dataset stands as one of the largest and most well-acknowledged open-source epilepsy EEG datasets available to researchers, offering detailed clinical case descriptions (Shah et al., 2018). It includes annotations on the timing and types of epileptic seizures, as well as comprehensive patient information such as sex, age, medications, clinical history, seizure event count, and duration. Our study utilized the May 2020 release of the corpus (V1.5.2), comprising 3,050 seizure cases across eight distinct seizure types, recorded at various sampling frequencies and montages. The seizure types include Focal Non-Specific Seizure (FNSZ), Generalized Non-Specific Seizure (GNSZ), Absence Seizure (ABSZ), Complex Partial Seizure (CPSZ), Tonic Clonic Seizure (TCSZ), Tonic Seizure (TNSZ), Simple Partial Seizure (SPSZ), and Myoclonic Seizure (MYSZ), as detailed in Table 1. Due to the limited number of MYSZ events, we excluded this type and focused on the remaining seven seizure categories for analysis.

**Table 1:** The statistics description of the TUSZ dataset.

Seizure type	Seizure Events	Patients	Duration (s)
FNSZ	1836	150	121,139
GNSZ	583	81	59,717
CPSZ	367	41	36,321
ABSZ	99	12	852
TNSZ	62	3	1,204
SPSZ	52	3	2,146
TCSZ	48	14	5,548
MYSZ	3	2	1,312

### 2.2 Preprocessing

The dataset contains an insufficient number of instances to effectively train the DL model. Therefore, we need a data augmentation scheme that can help us in increasing the amount of training data. To overcome this problem, we propose two data augmentation schemes for training our model. The raw full length EEG segment is split into several small signals using a fixed-size window. The splitting strategy is adopted by the training EEG set and the test EEG set. Every small signal is used as an individual instance to learn or test the proposed model. The division of an EEG signal into smaller segments is a standard procedure utilized in previous approaches (Ullah et al., 2018).

One scheme (scheme-1) involves choosing a non-overlapping window size of 512. Take the Bonn dataset for example, each signal of length 4,097 is segmented into 8 smaller signals, discarding the last data point. The small EEG signals of each class are divided into disjoint training and testing sets, which consist of 90 and 10% of total signals, respectively. In this way, a total of 800 instances are created for each category, 720 for training and 80 for testing.

The other scheme (scheme-2) is employed to further increase the amount of training data, generating an additional training dataset by adding a small amplitude of noise based on scheme-1. The procedure of generating additional training data set is as follow:


(1) x=s+α×σ×n

where *x* represents the generated additional training data; *s* represents the raw training EEG signal with a standard deviation of σ ; *n* represents the noise signal with a mean of 0 and a variance of 1; α represents the intensity of noise signal, and is set to 0.01. For the Bonn dataset, the artificial generate training data are created twice, and the testing data is the same as scheme-1. In this way, a total of 2,240 instances are created for each category, 2,160 for training and 80 for testing. For the TUSZ dataset, the artificially generated training data volume matches that of the original EEG training data.

Furthermore, for the TUSZ dataset, we employed the IBM TUSZ data-preparation version for building data (Roy et al., 2020), which utilizes the transverse central parietal montage (TCP) featuring 20 selected paired channels as input. Additionally, all EEG recordings were resampled to a uniform frequency of 250 Hz.

### 2.3 Residual and bidirectional LSTM

**Figure 1:**
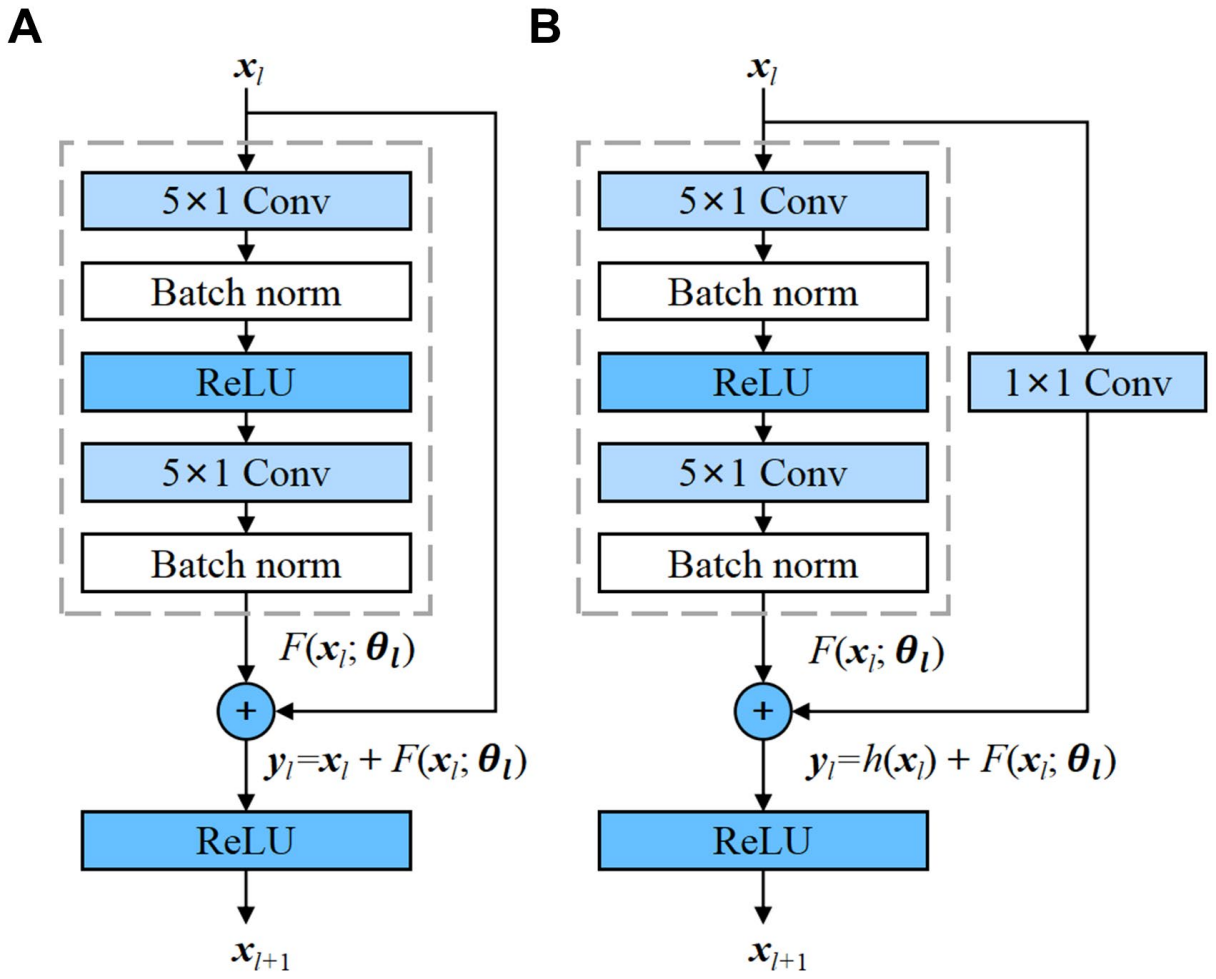
Residual blocks with and without convolution, which transforms the input into the desired shape for the addition operation. **(A)** Residual block without convolution; **(B)** Residual block with convolution.

As an emerging DL network structure, residual neural network (ResNet) has a deeper network and can achieve a better performance (He et al., 2016). The advantage of ResNet is that it solves the problem of gradient disappearance as the number of model layers increases. This is achieved by adding skip connections that bypass one or more layers in the network, allowing the input to be added directly to the output of a later layer. The residual block is the basic component of ResNet, and 1D residual blocks are used in the propose model as depicted in the Figure 1.

The residual block contains two convolutional layers, and each convolutional layer is followed by a batch normalization layer and a ReLU activation function. Then, a shortcut is used to skip these two convolution operations and add the input directly before the final ReLU activation function. Figure 1A demonstrates the presence of an identity mapping within the residual element, permitting the direct transmission of the input feature map to the output when the shape of the feature maps are identical. However, when the shape of the input and output feature maps differ, an additional 1 × 1 convolutional layer is required to transform the input for the addition operation, as depicted in Figure 1B. Each residual block can be expressed in a general form (He et al., 2016):


(2) $$y_{l}=h\left(x_{l}\right)+F\left(x_{l};\theta_{l}\right)$$

(3) $$x_{l+1}=f\left(y_{l}\right)$$

where *x_l_* and *x*_*l* + 1_ represent input and output of the *l*-th residual block, *F* is a residual function, $\theta_{l}$ includes all learnable parameters in the *l-*th residual block, and *f* is a ReLU function. In Figure 1A, *h*(*x_l_*) = *x_l_* is an identity mapping. In Figure 1B, *h*(*x*) is convolutional function. The residual blocks with and without convolution are adopted in the proposed model.

**Figure 2:**
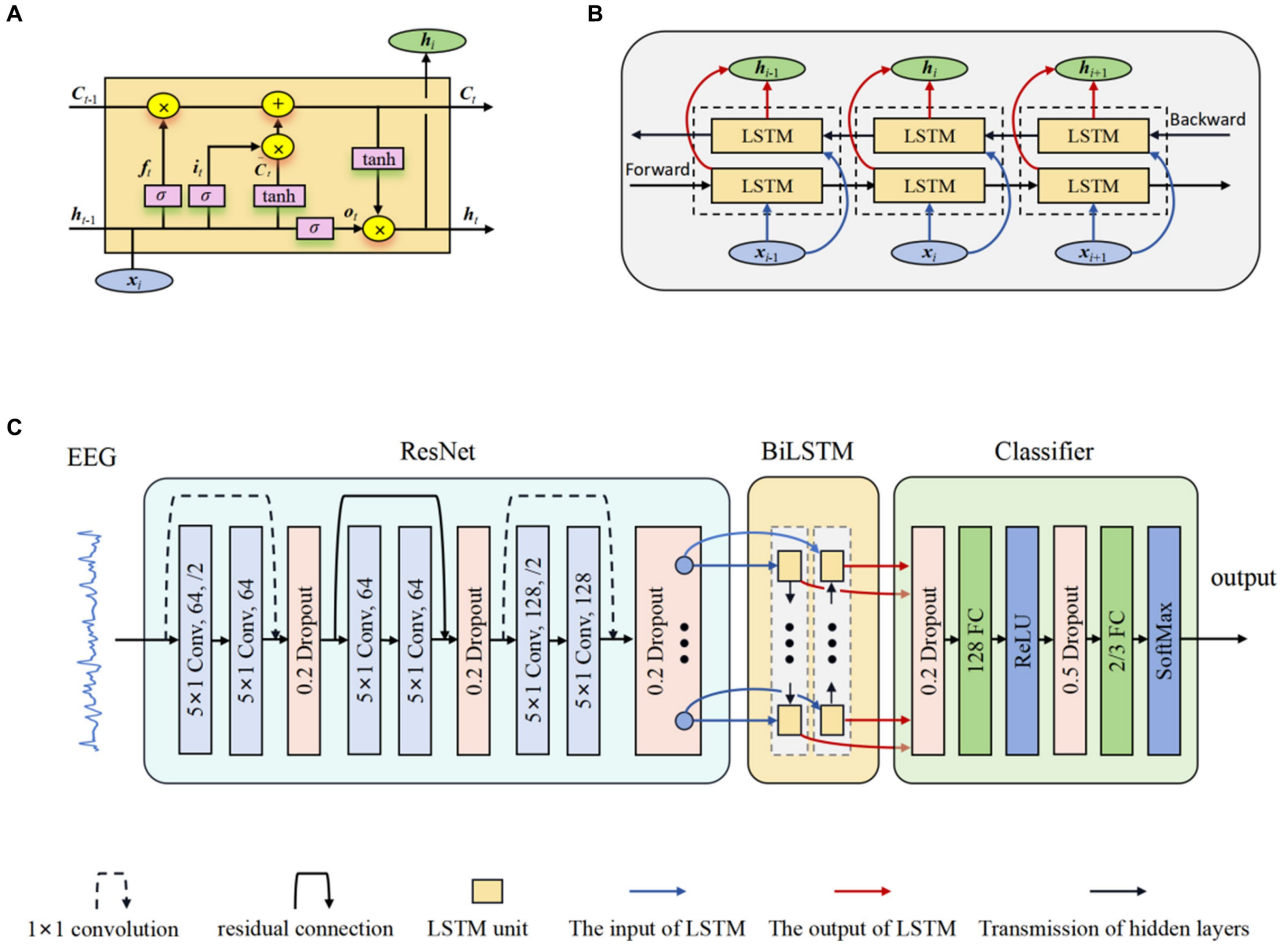
The structure of ResBiLSTM. **(A)** LSTM unit; **(B)** BiLSTM network; **(C)** Overall model architecture.

LSTM networks (Tuncer and Bolat, 2022b) were developed as an evolution over the RNNs, explicitly engineered to capture and retain long-term temporal dependencies. The innovative part of LSTM networks compared to traditional RNNs is the inclusion of gate mechanisms, namely the input gate, forget gate, and output gate, which allows the model to control more precisely what information needs to be kept in its memory cell and what needs to be removed. The input gate *i_t_* regulates the inflow of new information into the memory cell, allowing the network to determine the relevance and significance of incoming data. In contrast, the forget gate *f_t_* controls the extent to which previously stored information should be discarded from the cell’s memory. Finally, the output gate *o_t_* determines the flow of information from the memory cell, thereby regulating the contribution of the memory cell to the overall network output. The typical structure of an LSTM unit is depicted in Figure 2A. The formulas of the LSTM are explained as follows (Graves and Schmidhuber, 2005):


(4) $$f_{t}=\sigma \left(W_{f}\left[h_{t-1},x_{t}\right]+b_{f}\right)$$

(5) $$i_{t}=\sigma \left(W_{i}\left[h_{t-1},x_{t}\right]+b_{i}\right)$$

(6) $$o_{t}=\sigma \left(W_{o}\left[h_{t-1},x_{t}\right]+b_{o}\right)$$

(7) $${\tilde{C}}_{t}=\tanh\left(W_{C}\left[h_{t-1},x_{t}\right]+b_{C}\right)$$

(8) $$C_{t}=f_{t}\times C_{t-1}+i_{t}\times {\tilde{C}}_{t}$$

(9) $$h_{t}=o_{t}\times \tanh\left(C_{t}\right)$$

where *σ* (sigmoid) and tanh are activation functions; W_*_ are weight parameters; b_*_ are bias parameters; *h*_*t*-1_ and *h_t_* represent the previous and current hidden states; ${\tilde{C}}_{t}$ and *C_t_* represent candidate and current memory cell state, respectively.

BiLSTM networks (Graves and Schmidhuber, 2005) serve as a significant extension of the LSTM architecture, enabling the incorporation of information from both preceding and subsequent time steps. This is achieved through the utilization of two LSTM blocks, allowing the model to capture contextual dependencies in both temporal directions. This bidirectional flow significantly enhances the network’s capacity to capture comprehensive patterns and dependencies in sequential data, rendering it particularly effective for tasks demanding a nuanced analysis of temporal dynamics and complex sequential data. Figure 2B shows the internal structure of the BiLSTM networks. The hidden state used in BiLSTM is as follows:


(10) $$h_{t}^{\mathrm{BiLSTM}}=h_{t}^{\mathrm{forward}}⊕h_{t}^{\mathrm{backward}}$$

The proposed model for Epilepsy detection based on residual and BiLSTM network. Firstly, the ResNet actively learns the local correlation characteristics through the utilization of three residual blocks. Moreover, the learned features are fed into BiLSTM network to model the temporal dependencies. Subsequently, the extracted high-level epileptic features are inputted into a straightforward classifier module comprising two fully connected (FC) layers. To show the effectiveness of the proposed model, eight models with different configurations were designed. These models will guide the design of future models to better address overfitting. Table 2 shows the detailed specifications of these models. The detail of a ResBiLSTM model(*M*5) is depicted in Figure 2C. In order to streamline the architectural representation, the introduction of the residual block simplified drawing. For a more comprehensive understanding of its intricate arrangement, please refer to Figure 1, which illustrates the detailed structure of this process. The EEG recordings are directly used as the input of the proposed model, and the shape of the input data is 512 × *ch*, where *ch* represents the number of the raw EEG recording channel. The ResNet consists of three residual blocks, with each block being accompanied by a dropout layer incorporating a dropout rate of 0.2. This design choice aims to address the issue of overfitting, which can be mitigated through the application of dropout techniques. Within each residual block, every convolutional layer employs a convolution kernel with dimensions of 5 × 1. The first convolutional layer in the initial residual block has a stride of 2 and is equipped with 64 kernels. Additionally, the second convolutional layer in the same block has a stride of 1 and is also furnished with 64 kernels. The connection between the input and output of the initial residual block is established via a convolution operation, visually indicated by a dashed line in Figure 2C. The second and third residual blocks follow a similar procedure as the first residual block. The BiLSTM employs 64 neurons. The resulting output from the BiLSTM is then fed to a classifier composed of two dropout layers, two FC layers, and two activation layers. The first FC layer (FC1) integrates 128 neurons and is followed by a ReLU activation layer. The subsequent FC layer (FC2) matches the number of neurons to the total classes in the classification task. A softmax activation layer follows FC2 layer. Dropout layers, with probabilities of 0.2 and 0.5 respectively, are employed before each FC layer to mitigate overfitting.

**Table 2:** The specifications of eight models using 10-fold CV for fine tuning.

Layers\Model		*M*1	*M*2	*M*3	*M*4	*M*5	*M*6	*M*7	*M*8
Residual block 1	Number of kernels	32	32	32	32	64	64	64	64
	Kernel size	5	5	5	5	5	5	5	5
	Stride	2	2	2	2	2	2	2	2
Dropout 1		0.2	0.2	0.2	0.2	0.2	0.2	0.2	0.2
Residual block 2	Number of kernels	32	32	32	32	64	64	64	64
	Kernel size	5	5	5	5	5	5	5	5
	Stride	1	1	1	1	1	1	1	1
Dropout 2		0.2	0.2	0.2	0.2	0.2	0.2	0.2	0.2
Residual block 3	Number of kernels	64	64	64	64	128	128	128	128
	Kernel size	5	5	5	5	5	5	5	5
	Stride	2	2	2	2	2	2	2	2
Dropout 3		0.2	0.2	0.2	0.2	0.2	0.2	0.2	0.2
BiLSTM		64	64	128	128	64	64	128	128
Dropout		0.2	0.2	0.2	0.2	0.2	0.2	0.2	0.2
FC1		128	256	128	256	128	256	128	256
Dropout		0.5	0.5	0.5	0.5	0.5	0.5	0.5	0.5
FC2(Out)		2/3	2/3	2/3	2/3	2/3	2/3	2/3	2/3
Parameters		133,571	150,467	282,051	315,331	314,755	331,651	496,003	529,283

### 2.4 Strategy for the cross-validation and performance metrics

To evaluate our model, we implemented cross-validation (CV) to ensure thorough testing across various data variations. Specifically, we employed 10-fold CV for the Bonn dataset, 5-fold and 10-fold CV for the TUSZ dataset. Taking the Bonn dataset as an example, EEG signals for each class were randomly divided into 10 equal-sized folds. For each iteration, 1 fold (10%) served as the test set, while the remaining 9 folds (90%) were used for training the model. The CV process is then repeated 10 times, with each of the 10 fold used exactly once as the test data. The average performance is calculated for 10 folds. We used evaluation criteria commonly used in classification to measure the validity and robustness of the proposed model from different perspectives, including accuracy, precision, recall, F1-score and weighted F1-score. These indicators are defined as:


(11) $$\mathrm{Accuracy}\;\left(Acc\right)=\frac{TP+TN}{TP+TN+FP+FN}$$

(12) $$\mathrm{Precision}\;\left(Pre\right)=\frac{TP}{TP+FP}$$

(13) $$\mathrm{Recall}\;\left(Rec\right)=\frac{TP}{TP+FN}$$

(14) $$F1-\mathrm{score}\left(F1\right)=2\times \frac{\mathrm{Precision}\times \mathrm{Recall}}{\mathrm{Precision}+\mathrm{Recall}}$$

(15) $$\mathrm{Weighted}\;F1=\sum_{k=1}^{K}\omega_{k}F1_{k}$$

where *TP* and *TN* are symbols of the correct positive sample number and the correct negative sample number predicted by the model, respectively. *FP* and *FN* are symbols of the false positive sample number and the false negative sample number predicted by the model, respectively. F1-score is a comprehensive indicator that measures the classification performance. A larger values of these indicators mean better classification performance. The weighted *F*1 is under the proportion ω*^i^* of the *i*-th seizure type, and K denotes the total number of all attack types. The weighted F1-score is calculated in proportion $\omega_{i}$ to the *i*-th seizure type, where *K* represents the total number of seizure categories.

The ResBiLSTM model’s weight parameters are learned using traditional back-propagation, with the cross-entropy function as the loss function and the Adam optimizer for optimization. For the Bonn dataset, settings include a learning rate of 0.0001, batch size of 64, and 100 training epochs. For the TUSZ dataset, the learning rate is set to 0.001, with a batch size of 320 and 200 training epochs. We select the model’s best weight parameters based on optimal accuracy and minimal loss across all training iterations. All DL models were developed using Pytorch, an open-source DL framework. A Debian 11 operating system-based workstation equipped with an Intel Core i9-9820X CPU and RTX3090 GPU facilitated the model’s training and testing.

## 3 Model performance and comparisons

### 3.1 Performance in fine tuning

For optimal model selection, we considered eight ResBiLSTM models in our initial experiments, as is shown in Table 2. For selecting the best model, we need to address two questions: (a) How can the hyper-parameters of the model be optimized to enhance its generalization capability and suitability for diverse epilepsy detection tasks? (b) Which data augmentation scheme is the most appropriate?

To address these inquiries, we conducted comprehensive experiments on the models, employing 10-fold CV for three type classification tasks on the Bonn dataset: (i) non-seizure vs. seizure (two-category: D-E); (ii) healthy vs. interictal vs. ictal (three-category: AB-CD-E), (iii) eyes opened vs. eyes closed vs. interictal out the epileptogenic zone vs. interictal within the epileptogenic zone vs. seizure ictal (five-category: A-B-C-D-E) epilepsy detection tasks. The model’s hyper-parameters, including the count of kernels in residual blocks, BiLSTM units, and FC1 layer neurons, were adjusted during experiments, resulting in eight different models. Increasing the number of convolutional kernels, BiLSTM units, and neurons in the classifier typically enhances the model’s expressive power, thereby improving its ability to learn complex patterns in the EEG signal. However, this also increases computational cost and the risk of overfitting, especially when training data is limited. An appropriate number of convolutional kernels and BiLSTM units can effectively capture spatio-temporal features while mitigating overfitting. To find the balance between model complexity and performance, we conducted extensive preliminary experiments. The number of kernels in each residual block of models *M*5 to *M*8 is twice that of *M*1 to *M*4, respectively. The two convolutional layers within each residual block employ an identical number of convolutional kernels. The number of BiLSTM units of models *M*1, *M*2, *M*5, and *M*6 are equal that is 64, and that is 128 in models *M*3, *M*4, *M*7, and *M*8. The number of neurons in the FC1 layer of models *M*1, *M*3, *M*5, and *M*7 are equal that is 128, and that is 256 in models *M*2, *M*4, *M*6, and *M*8. The models are trained and tested using data augmentation scheme-1 and scheme-2. The detailed specifications of these models and their corresponding parameter quantities are presented in Table 2. The values of the hyper-parameters in Table 2 are based on preliminary experimental experiences. The optimal model and data augmentation scheme from these experiments are applied to other epileptic seizure classification tasks.

**Figure 3:**
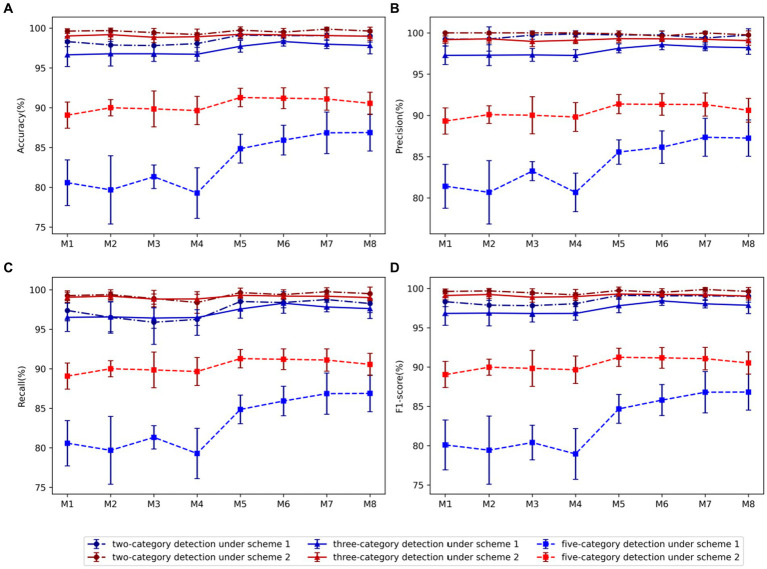
Comparison of evaluation metrics of each model using two data augmentation schemes: **(A)** Accuracy, **(B)** Precision, **(C)** Recall, **(D)** F1-score. Among them, blue represents scheme-1, red represents scheme-2, dot-dash line and dot represents two-category detection, solid line and triangle represents three-category detection, and dash line and square represents five-category detection.

The performance disparities among these eight models are illustrated in Figure 3. It can also be clearly seen that the performance of scheme-2 is better than scheme-1. For two-category, three-category, and five-category classifications, the average accuracy of augmentation scheme-2 surpass those of augmentation scheme-1 by 1.05, 1.70, and 7.17%, respectively. Almost similar results can be observed for other performance metrics. Therefore, scheme-2 is adopted in all other experiments in this study.

Using Scheme-2, we conducted a comparative analysis between models *M*1-*M*4 and models *M*5-*M*8, revealing that in the case of the two-category classification task, the variation in the number of kernels in the residual block does not significantly impact performance. However, for the three-category and five-category classification tasks, an increase in the number of kernels within the residual block markedly enhances model performance. Consequently, we proceed to select the optimal model from the models *M*5-*M*8. In the comparison between *M*5 and *M*6 utilizing 64 BiLSTM neurons and *M*7 and *M*8 employing 128 neurons, the findings indicated superior performance for the 128 BiLSTM neuron model in the two-category classification tasks. However, in the three-category and five-category classification tasks, the 64-neuron model exhibited superior performance. Upon comparing models *M*5 and *M*7, which utilize 128 neurons in the FC1 layer, with models M6 and M8 employing 256 neurons, it is evident that the model with 128 neurons performs better in identifying the five classification tasks. Overall, model *M*5 has the best average performance, with accuracy, precision, recall and F1 score of 96.75, 96.85, 96.73, and 96.76, respectively.

To further optimize hyper-parameters, we conducted a comparative study on network depth. Using model M5 as a base, we adjusted either the number of residual blocks in the ResNet or the number of layers in the BiLSTM network. When reducing the depth of the ResNet module, we removed the corresponding residual blocks from model M5. For the configuration with 4 residual blocks, we added an additional residual block after the third block of model M5, with 128 kernels, a kernel size of 5 × 1, and a stride of 1. For the configuration with 5 residual blocks, we further added another residual block with 256 kernels, a kernel size of 5 × 1, and a stride of 2. When increasing the depth of the BiLSTM network, the number of neurons per layer remained consistent. The experimental results are presented in Table 3. From Table 3, it is evident that models with a certain depth of residual networks perform better, while different depths of BiLSTM do not show a significant impact on model performance. For the binary classification task of detecting epileptic seizures, the model achieved optimal performance with 5 residual blocks and a single-layer BiLSTM, attaining an accuracy of 99.88%, precision of 100.00%, recall of 99.75%, and F1 score of 99.87%. For the three-category classification task, the best performance was achieved with 4 residual blocks and one layer BiLSTM, with an accuracy of 99.23%, precision of 99.34%, recall of 99.31%, and F1 score of 99.32%. For the most challenging five-class classification task, the optimal configuration was 3 residual blocks and a two-layer BiLSTM, achieving an accuracy of 91.60%, precision of 91.76%, recall of 91.60%, and F1 score of 91.57%.

**Table 3:** Performance of models with various network layer configurations.

Configuration	Residual Block	1	2	3	4	5	3	3
	BiLSTM Layers	1	1	1	1	1	2	3
Two-category	Accuracy	98.56	99.31	99.75	99.75	**99.88**	99.75	99.56
	Precision	99.01	99.50	99.88	99.88	**100.00**	99.88	99.75
	Recall	98.13	99.13	99.63	99.63	**99.75**	99.63	99.38
	F1-Score	98.56	99.31	99.75	99.75	**99.87**	99.75	99.56
Three-category	Accuracy	97.48	98.58	**99.23**	**99.23**	98.98	98.90	99.03
	Precision	97.78	98.71	99.30	**99.34**	99.13	99.03	99.19
	Recall	97.46	98.67	99.29	**99.31**	98.98	98.88	99.08
	F1-Score	97.60	98.68	99.29	**99.32**	99.05	98.95	99.12
Five-category	Accuracy	77.98	85.78	91.28	90.93	90.85	**91.60**	90.85
	Precision	78.64	86.15	91.38	91.02	91.02	**91.76**	91.01
	Recall	77.98	85.78	91.28	90.93	90.85	**91.60**	90.85
	F1-Score	77.41	85.66	91.24	90.93	90.83	**91.57**	90.82

The bold font highlights the best result among the different methods.

Deeper models have more learnable parameters, which can lead to overfitting when there is insufficient training data. As shown clearly in Table 3, while increasing the network depth can improve the model’s performance in specific epilepsy detection tasks, it can also lead to a decline in performance for other detection tasks. Therefore, considering the model size, recognition accuracy, and generalization ability, we selected the M5 model, configured with 3 residual blocks and one layer BiLSTM, as the optimal model for subsequent epilepsy detection experiments and comparative analysis with other approaches.

### 3.2 Comparisons with the baseline models

**Figure 4:**
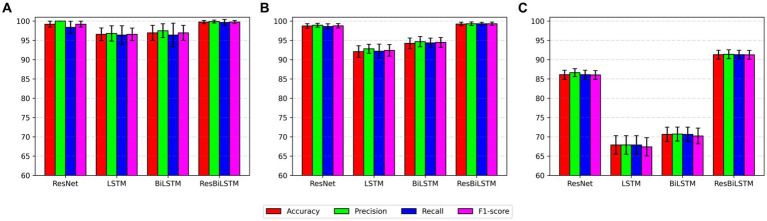
Comparison of performance with baseline approaches: **(A)** two-category, **(B)** three-category, **(C)** five-category.

The empirical analysis is compared with baseline approaches, including ResNet, LSTM, and BiLSTM, across two-category, three-category, and five-category epilepsy detection tasks. Figure 4 illustrates the ResBiLSTM model’s dominance, followed by the ResNet model, with the BiLSTM and LSTM models showing comparatively lower performance. In epilepsy onset detection, all methods surpassing 96% in epilepsy onset detection. The ResBiLSTM performed the best, followed by the ResNet model. The ResNet model consistently outperforms the BiLSTM and LSTM models by over 2% across all measures. In the case of the complex three-category classification task, the ResBiLSTM model demonstrates superior performance, surpassing the ResNet, BiLSTM, and LSTM models by 0.49, 4.99, and 7.15% in accuracy, respectively. The ResBiLSTM model exhibits a similar advantage across other performance metrics. In the intricate five-category classification problem, the ResBiLSTM model exhibits distinct advantages over other models, leading by margins of 4.76–23.84% across various metrics. The funding shows that the increasing complexity of the recognition tasks accentuates the superiority of the ResBiLSTM model.

**Figure 5:**
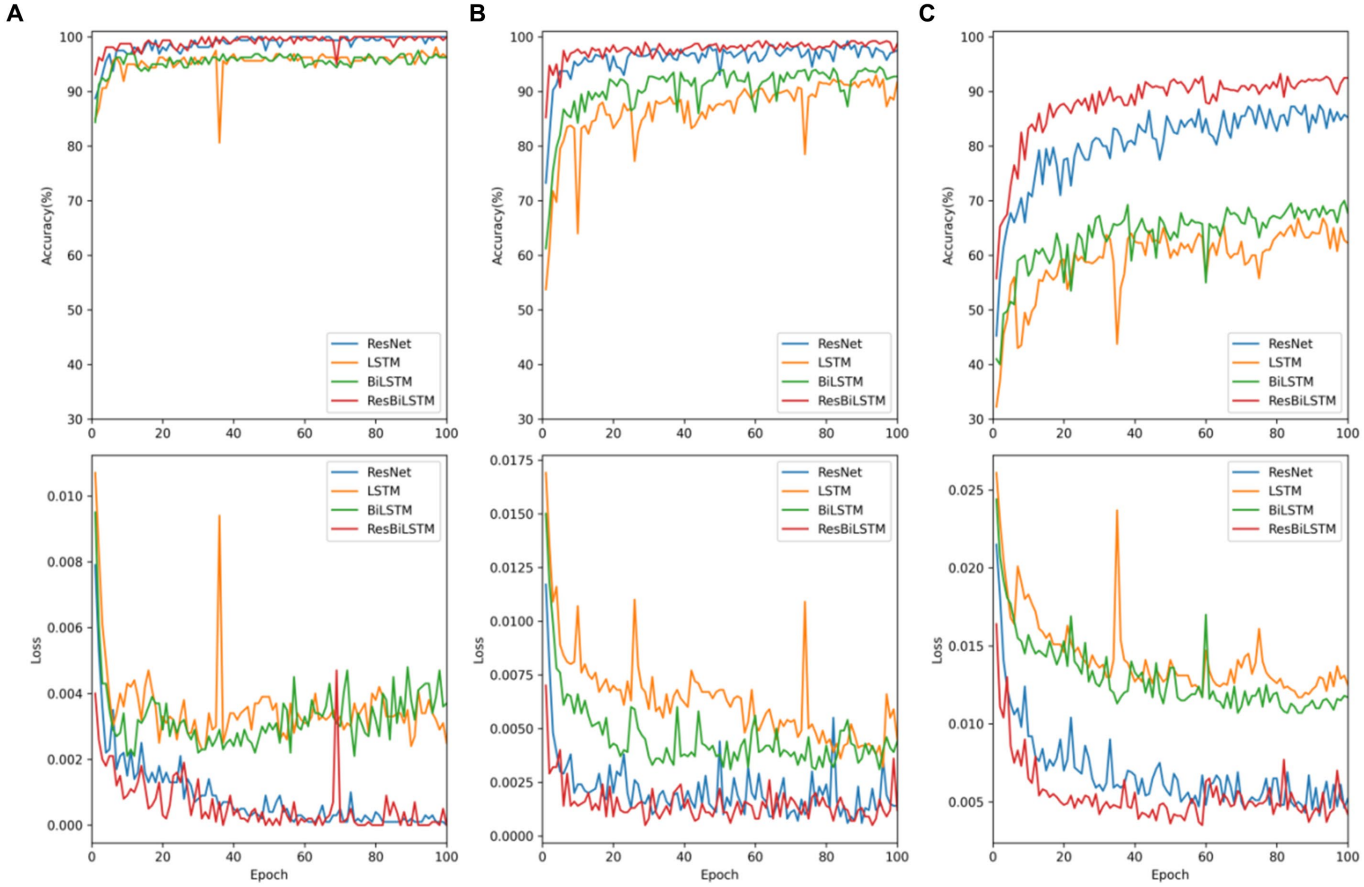
Testing accuracy and loss of these models on recognition tasks: **(A)** two-category, **(B)** three-category, **(C)** five-category.

To comprehensively assess the performance disparities among various methods during the testing procedure, Figure 5 exhibits the test accuracy and loss of the four methods throughout the initial-fold CV testing. The two-category classification performance comparison is displayed in Figure 5A. Both the ResBiLSTM model and ResNet model exhibit excellent performance, demonstrating similar accuracy close to 100%. In the three-category classification comparison depicted in Figure 5B, the ResBiLSTM model outperforms the ResNet model slightly and significantly surpasses the BiLSTM and LSTM models. Additionally, the BiLSTM model demonstrates superior performance compared to the LSTM model. The five-category classification performance, as shown in Figure 5C, indicates that the ResBiLSTM achieves the highest accuracy across the entire testing process. Notably, the proposed model demonstrates a notably faster convergence rate compared to other methods.

**Figure 6:**
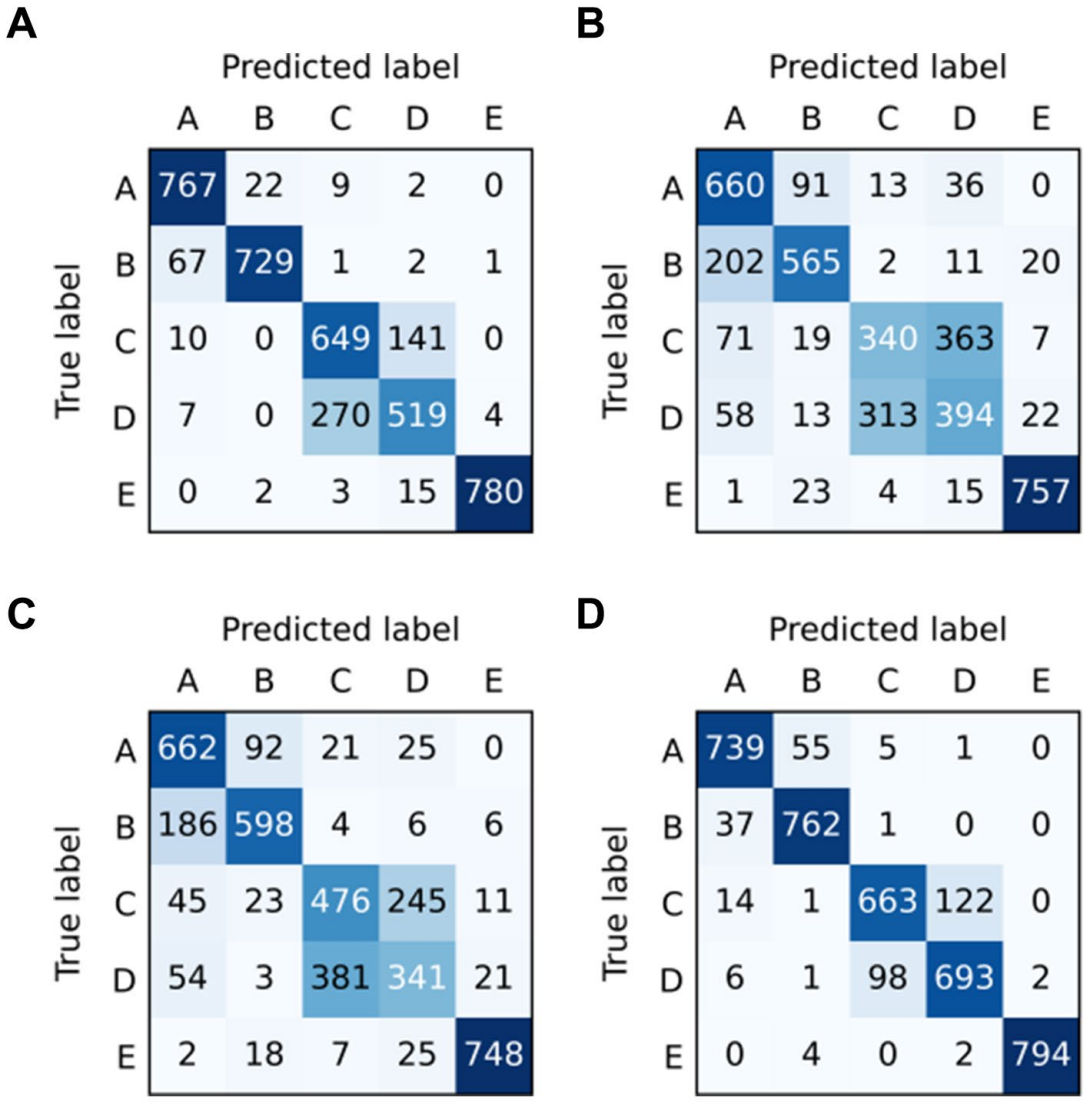
Confusion matrix of models in five-category detection task: **(A)** ResNet, **(B)** LSTM, **(C)** BiLSTM, and **(D)** ResBiLSTM.

To analyze the epileptic detection performance of each condition, the confusion of models ResNet, LSTM, BiLSTM, and ResBiLSTM are depicted in Figure 6. The row and column in these confusion matrices indicate the true labels and predicted labels. It can be seen that the primary source of error arises notably from the misclassification of the samples within sets C and D, subsequently followed by misclassifications within sets A and B. Furthermore, The misclassification of ResBiLSTM is relatively better than that of other approaches.

### 3.3 Comparison between various seizure detection tasks on Bonn dataset

We conducted three group experiment of epilepsy recognition, using the *M*5 model, and using data augmentation scheme-2: (i) two-category classification tasks (A-E, B-E, C-E, D-E, AB-E, CD-E, C-DE, ABCD-E), (ii) three-category classification tasks (A-C-E, A-D-E, B-C-E, B-D-E, AB-CD-E), (iii) five-category classification task (A-B-C-D-E).

**Figure 7:**
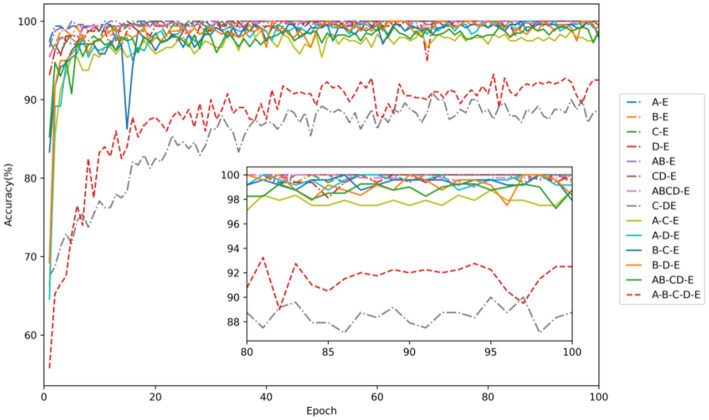
Testing accuracy in each task of EEG signal classification for epilepsy detection. Among them, dot-dash lines represent two-category classification, solid lines denote three-category classification, and a dashed line represents five-category classification.

Figure 7 demonstrates that the classification problem concerning the onset of epilepsy exhibits the fastest convergence speed and the highest accuracy. On the other hand, the accuracy of the two-category classification problem, aimed at detecting epileptic seizure regions (C-DE), requires improvement. The detailed average experimental performances are presented in Table 4. In the two-category epileptic seizure recognition tasks (A-E, B-E, C-E, D-E, AB-E, CD-E, ABCD-E) which are used to identify seizures, all evaluation indicators exceeded 99%. Notably, the A-E and C-E classification tasks achieved perfect scores of 100% across all evaluation metrics. However, for the C-DE classification task identifying epileptogenic zone, the accuracy, precision, recall, and F1-score are 89.42, 91.71, 92.50, and 88.04%, respectively. While the two-category classification experiment for identifying the presence of epilepsy seizures is relatively straightforward, distinguishing the epileptogenic zone proves to be more challenging due to the sets C and D are both collected from the interictal periods of epileptic patients. The three-category classification experiments aimed to distinguish healthy, interictal, and ictal. Notably, both B-C-E and B-D-E demonstrated better performance, with all indicators exceeding 99.45%. However, the model exhibited relatively weaker performance on the A-C-E classification task, with accuracy, precision, recall, and F1 values of 98.88, 98.89, 98.88, and 98.87%, respectively. Moreover, all average indicators in the five-category classification experiment surpassed 91.2%. The identification of five-category classification problems presents the greatest challenge.

**Table 4:** The average performance of all experiment tasks using 10-fold CV with model *M*5 (%).

Class labels	Accuracy	Precision	Recall	F1-score
A-E	100.00	100.00	100.00	100.00
B-E	99.88	99.88	99.88	99.87
C-E	100.00	100.00	100.00	100.00
D-E	99.75	99.88	99.63	99.75
AB-E	99.92	99.88	99.88	99.91
C-DE	89.42	91.71	92.50	88.04
CD-E	99.71	99.75	99.38	99.67
ABCD-E	99.83	99.88	99.25	99.73
A-C-E	98.88	98.89	98.88	98.87
A-D-E	99.04	99.06	99.04	99.04
B-C-E	99.46	99.47	99.46	99.46
B-D-E	99.46	99.47	99.46	99.46
AB-CD-E	99.23	99.30	99.29	99.29
A-B-C-D-E	91.27	91.38	91.27	91.24

**Figure 8:**
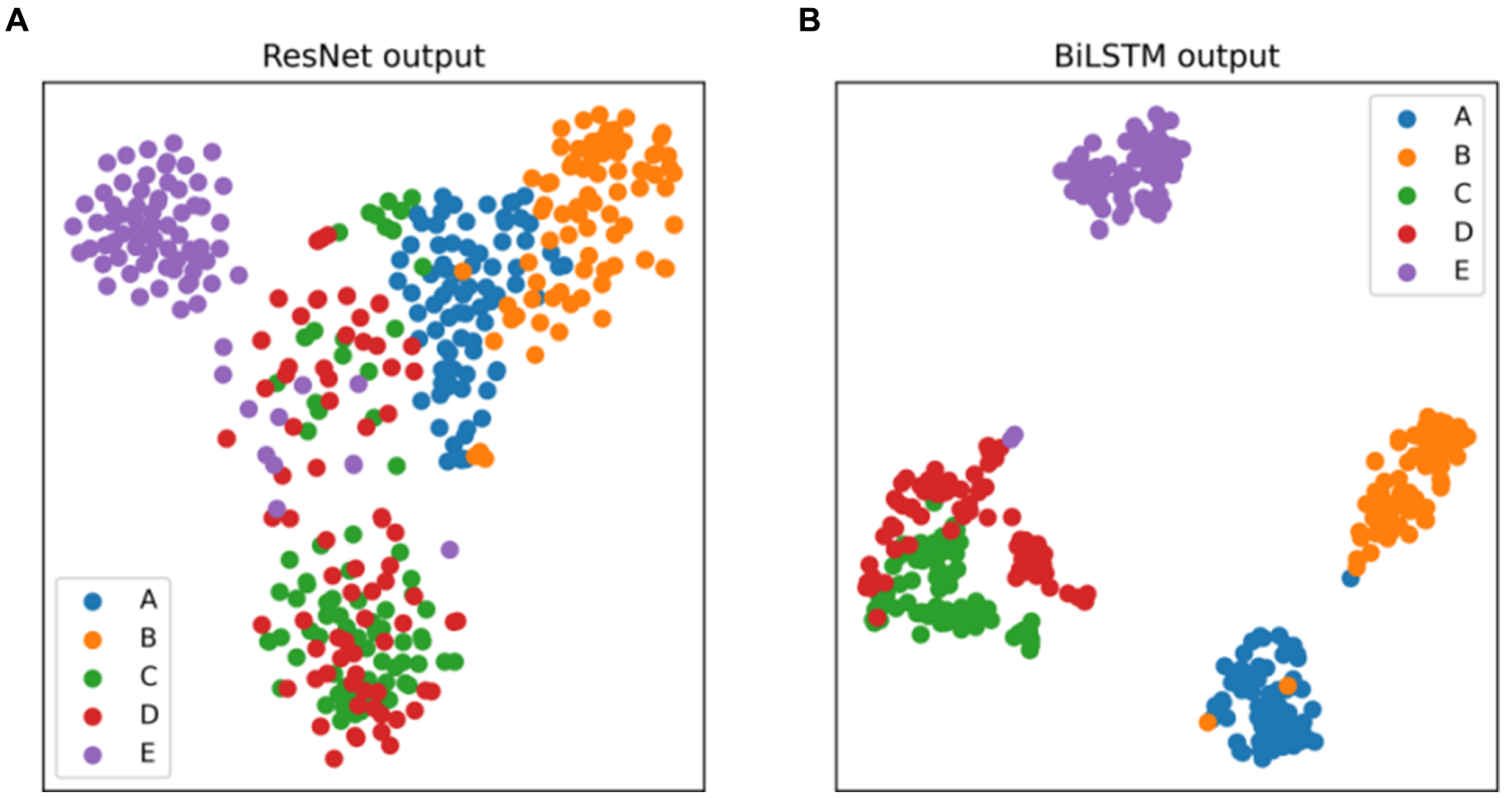
Two-dimensional t-SNE visualization of the features at the output layer of **(A)** ResNet, **(B)** BiLSTM.

The t-distributed stochastic neighbor embedding (t-SNE) serves as a widely-used statistical method for dimension reduction and visualization. Its adoption aids in gauging the discriminative nature of the extracted features. Figure 8 presents the t-SNE visualizations of the output features from the ResNet and BiLSTM module. Figure 8A demonstrates that the features extracted by the ResNet module from the EEG signals can preliminarily differentiate healthy (sets A and B), interictal (sets C and D), and ictal (set E) states. Notably, there exists considerable overlap between EEG sets C and D, both acquired from the interictal period. Moreover, Figure 8B demonstrates that the incorporation of BiLSTM module to capture the temporal dependencies results in the acquisition of more discriminative EEG features. It leads to a notable expansion in inter-category distances and a reduction in intra-category distances. These experimental findings collectively indicate the model’s ability to achieve exceptional recognition performance across various epilepsy detection tasks.

### 3.4 Seizure detection tasks on TUSZ dataset

**Figure 9:**
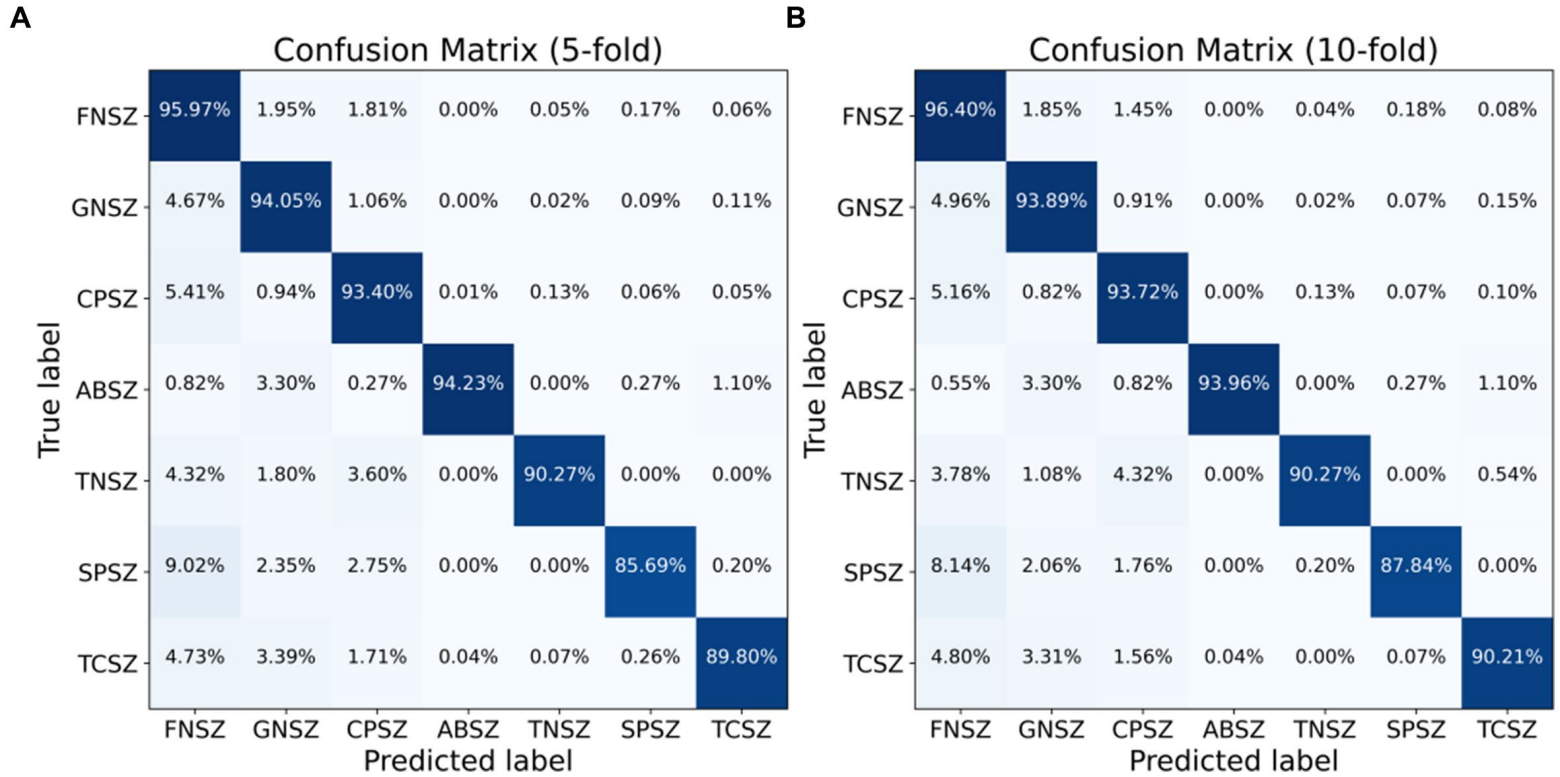
Classification confusion matrix for seven seizure types: **(A)** 5-fold CV, **(B)** 10-fold CV.

We employed both 5-fold and 10-fold CV strategies using the ResBiLSTM model to identify seven types of epileptic seizures on the TUSZ dataset. The confusion matrices of the classification results are shown in Figure 9, with Figure 9A depicting the 5-fold CV confusion matrix and Figure 9B depicting the 10-fold CV confusion matrix. From Figure 9A, it is evident that under the 5-fold CV conditions, the FNSZ seizure type achieved the highest recognition accuracy at 95.97%, while SPSZ had the lowest accuracy at 85.69%. The most severe misclassification was SPSZ being incorrectly classified as FNSZ, with a misclassification rate of 9.02%. From Figure 9B, under the 10-fold CV conditions, the average recognition accuracies for FNSZ, GNSZ, CPSZ, ABSZ, TNSZ, SPSZ, and TCSZ were 96.40, 93.89, 93.72, 93.96, 90.27, 87.84, and 90.21%, respectively. Compared to the 5-fold CV results, there were significant improvements in the recognition accuracies for SPSZ, FNSZ, and TCSZ, with increases of 2.15, 0.43, and 0.41%, respectively. The highest misclassification rate was still SPSZ being misclassified as FNSZ, with a misclassification rate of 8.14%. Overall, the average recognition accuracy and weighted F1 score for the ResBiLSTM model were 94.76 and 94.77% for the 5-fold CV, and 95.03 and 95.03% for the 10-fold CV, respectively, indicating that the 10-fold CV results were slightly better than those of the 5-fold CV.

## 4 Discussion

### 4.1 Limitations of previous studies and advantages of the ResBiLSTM model

Epileptic seizure detection algorithms are crucial for providing timely and accurate diagnosis, which is essential for effective treatment and management of epilepsy. These algorithms can significantly improve patient outcomes by enabling early intervention and reducing the risk of seizure-related complications. Traditional epileptic seizure detection algorithms typically rely on hand-crafted feature extractors combined with machine learning algorithms. The quality of these features is highly dependent on the expertise and experience of the designer. Since these feature extractors are tailored for specific datasets or tasks, their generalizability across different datasets or tasks is limited. Additionally, traditional methods separate feature extraction and classification, lacking end-to-end learning capabilities. This separation means the model cannot adjust the feature extraction process based on the final classification outcomes, thus limiting overall performance. To address these issues, several end-to-end DL-based algorithms for epileptic seizure detection have emerged. These algorithms primarily include CNN-based methods, RNN-based methods, and hybrid models combining both CNN and RNN. CNNs are highly effective at capturing and extracting spatial features from raw EEG data, eliminating the need for hand-crafted feature extractors. Due to the parallel computation nature of convolution operations, CNN models offer faster training and inference speeds. However, CNNs have limited ability to handle time-series data and capture the temporal dynamics of EEG signals, which may restricts their accuracy in epileptic seizure detection. RNNs, particularly LSTM networks and BiLSTM networks, excel at capturing temporal dynamics in sequential data. LSTM networks can capture long-term dependencies in EEG signals, which is crucial for epileptic seizure detection. Nonetheless, RNNs have high computational complexity, longer training times, and are challenging to parallelize. RNNs also encounter issues such as gradient vanishing and exploding when handling long sequences, though LSTM and BiLSTM mitigate these problems to some extent. To leverage the advantages of both CNNs and RNNs, hybrid models utilize the spatial feature extraction capabilities of CNNs along with the temporal feature capturing abilities of RNNs, providing a more comprehensive solution for epileptic seizure detection. Although current hybrid models excel in binary classification tasks for epileptic seizure detection, there is considerable potential for enhancing both recognition accuracy and generalization capability in multi-category classification tasks.

Inspired by these studies, we propose the ResBiLSTM model for epileptic seizure detection. ResBiLSTM first utilizes a ResNet to automatically extract local spatial features from EEG signals. Subsequently, a BiLSTM network captures the temporal dynamics within these EEG features. The ResNet addresses the vanishing gradient problem in deep networks by introducing skip connections, enabling the training of deeper networks and the extraction of richer features. The BiLSTM network, on the other hand, simultaneously considers the forward and backward dependencies in time series data, capturing long-term dependencies that are crucial for a comprehensive understanding of signal variations, which is particularly important for epileptic seizure detection. Experimental results demonstrate that the ResBiLSTM model effectively combines the strengths of CNNs and RNNs. Comparative analysis with baseline approaches such as ResNet, LSTM, and BiLSTM illustrates the superior performance of the proposed ResBiLSTM model across these tasks, with its increasing advantage becoming more prominent as the task complexity escalates. By enhancing the extraction of both spatial and temporal features, ResBiLSTM improves recognition accuracy and generalization ability, making it a highly effective method for epileptic seizure detection.

### 4.2 Evaluation of data augmentation strategies and model hyperparameter tuning

Using the Bonn dataset, we validated the efficacy of the proposed model across three distinct classification tasks: two-category, three-category, and five-category epilepsy detection tasks. Experimental results indicate that employing data augmentation scheme-2, which involves segmenting EEG signals and adding slight white noise, significantly enhances the performance of the ResBiLSTM model compared to using data augmentation scheme-1, which only involves non-overlapping segmentation. This improvement is attributed to the fact that scheme-2 generates a larger volume of training data. Consequently, for epileptic seizure detection, exploring more effective methods to generate extensive training datasets for better DL model training is a promising area of research.

Additionally, the experimental results for finding the optimal hyper-parameters of the model indicate that using more network nodes in ResBiLSTM enhances its expressive power, leading to higher recognition accuracy. However, having too many nodes can result in overfitting. For the binary classification task of detecting epileptic seizures, the model performs better with deeper residual layers. However, for the three-class and five-class classification tasks, a moderate depth of residual layers yields the best performance. Increasing the depth of the BiLSTM network did not show a consistent pattern in performance improvement. In summary, adjusting the network layer depth can indeed improve accuracy for specific classification tasks but may also reduce accuracy for others. Therefore, further fine-tuning of the network depth and the number of nodes might help identify an even better model configuration.

### 4.3 Comparison and interpretation of evaluation metrics

In our study, we employed multiple metrics to evaluate the performance of our ResBiLSTM model. For the Bonn epilepsy dataset, we used accuracy, precision, recall, and F1-score as evaluation metrics. For the TUSZ epilepsy seizure type detection dataset, which is highly imbalanced, we used accuracy and weighted F1-score as evaluation metrics. The experimental results demonstrate that our ResBiLSTM model performs exceptionally well across these evaluation metrics.

The Accuracy metric indicates the proportion of correctly predicted instances out of the total instances. While it provides a general measure of model performance, it can be misleading in imbalanced datasets where the number of instances in each class varies significantly. The precision metric measures the proportion of true positive predictions out of all positive predictions made by the model. High precision indicates that the model makes fewer false positive errors. The recall metric measures the proportion of true positive predictions out of all actual positive instances. High recall indicates that the model successfully identifies most of the positive instances, which is critical in medical diagnosis scenarios such as epileptic seizure detection, as missed diagnoses (false negatives) can have severe consequences. The F1-score is the harmonic mean of precision and recall, providing a single metric that balances both. It is particularly useful when the importance of precision and recall needs to be balanced. The weighted F1-score takes into account the support (the number of true instances) for each class, calculating the F1-score for each class independently and then computing their weighted average. This metric is especially useful for imbalanced datasets as it provides a more comprehensive measure of overall model performance.

For the Bonn epilepsy dataset, the combination of accuracy, precision, recall, and F1 score provides a holistic view of the model’s performance. Precision and recall are particularly important in medical diagnostics, where false positives and false negatives have significant implications. Compared to the baseline methods (ResNet, LSTM, and BiLSTM), the ResBiLSTM model exhibits superior performance across all four metrics. Moreover, these advantages become increasingly pronounced as the classification tasks grow more complex. For the TUSZ dataset, the use of the weighted F1 score is crucial due to the dataset’s imbalance. The weighted F1 score ensures that the model’s performance is not biased towards the majority class and provides a balanced evaluation across all classes. Our model’s high weighted F1-score indicates its robustness and reliability in handling imbalanced data, which is common in real-world medical applications.

In summary, the comprehensive evaluation using these metrics shows that the ResBiLSTM model not only achieves high overall accuracy but also excels in reducing errors and maintaining balanced performance across classes, making it a reliable tool for clinical applications.

### 4.4 Comparison with other state-of-the-art approaches

Comparative experiments were conducted between the proposed model and several recent state-of-the-art approaches for epileptic seizure detection. A total of 14 recognition tasks on Bonn dataset were performed, comprising eight two-category tasks, five three-category tasks, and one five-category task. Table 5 presents a comparison with recent DL approaches from the literature, including an RNN-based method (Tuncer and Bolat, 2022b), and several hybrid-based methods (Xu et al., 2020; Ahmad et al., 2023; Pandey et al., 2023; Qiu et al., 2023; Shanmugam and Dharmar, 2023; Wang et al., 2023).

**Table 5:** The comparison between the ResBiLSTM and other recent DL state-of-the-art methods using the Bonn dataset.

Class labels	Publication year and author	Methodology	Acc(%)	Our Acc(%)
A-E	Tuncer and Bolat (2022b)	BiLSTM	**100**	**100**
	Wang et al. (2023)	CNN + LSTM	**100**	
	Qiu et al. (2023)	ResNet + LSTM	**100**	
B-E	Tuncer and Bolat (2022b)	BiLSTM	**100**	99.88
	Wang et al. (2023)	CNN + LSTM	**100**	
	Qiu et al. (2023)	ResNet+LSTM	**100**	
C-E	Tuncer and Bolat (2022b)	BiLSTM	**100**	**100**
	Wang et al. (2023)	CNN + LSTM	98.2	
	Qiu et al. (2023)	ResNet + LSTM	99.78	
D-E	Tuncer and Bolat (2022b)	BiLSTM	96	**99.75**
	Wang et al. (2023)	CNN + LSTM	97.6	
	Ahmad et al. (2023)	CNN + BiLSTM	99.41	
	Qiu et al. (2023)	ResNet + LSTM	99.57	
AB-E	Xu et al. (2020)	CNN + LSTM	99.39	**99.92**
	Wang et al. (2023)	CNN + LSTM	98.3	
C-DE	Tuncer and Bolat (2022b)	BiLSTM	**100**	89.42
CD-E	Wang et al. (2023)	CNN + LSTM	97.9	**99.71**
ABCD-E	Tuncer and Bolat (2022b)	BiLSTM	96	**99.83**
	Wang et al. (2023)	CNN + LSTM	98.7	
A-C-E	Shanmugam and Dharmar (2023)	CNN + LSTM	97.43	98.88
	Pandey et al. (2023)	CNN + LSTM	**99.33**	
A-D-E	Shanmugam and Dharmar (2023)	CNN + LSTM	97.36	**99.04**
B-C-E	Shanmugam and Dharmar (2023)	CNN + LSTM	99.09	**99.46**
B-D-E	Shanmugam and Dharmar (2023)	CNN + LSTM	99.37	**99.46**
AB-CD-E	Tuncer and Bolat (2022b)	BiLSTM	88	**99.23**
	Wang et al. (2023)	CNN + LSTM	98	
	Shanmugam and Dharmar (2023)	CNN + LSTM	97.08	
	Qiu et al. (2023)	ResNet+LSTM	98.17	
A-B-C-D-E	Xu et al. (2020)	CNN + LSTM	82	91.27
	Shanmugam and Dharmar (2023)	CNN + LSTM	**92.5**	
	Ahmad et al. (2023)	CNN + BiLSTM	84.1	
	Qiu et al. (2023)	ResNet + LSTM	90.17	

The bold font highlights the best result among the different methods.

As shown in Table 5, our proposal achieves best results in two-category classification tasks (A-E, C-E, D-E, AB-E, CD-E, ABCD-E). In the B-E classification task, our proposed method demonstrates a slightly lower accuracy of 99.88% compared to other methods. Notably, In the C-DE classification task, aimed at identifying the epileptogenic zone, the method in Tuncer and Bolat (2022b) outperforms ours by 10.58% in accuracy, while our model surpasses it by 11.23% in the AB-CD-E three-classification task. It is important to note that Tuncer and Bolat (2022b) only conducted experiments for binary and three-category classifications, where their model showed high performance in specific binary tasks. In contrast, our ResBiLSTM model demonstrated robust performance not only in binary and three-category classifications but also in more complex five-class tasks. Two factors may contribute to the observed discrepancy in accuracy between our study and Tuncer et al.’s study. Firstly, Tuncer and Bolat (2022b) used a feature extraction approach involving instantaneous frequency and spectral entropy from EEG signals, which are effective in capturing the underlying characteristics of epileptic activity. These features are specifically designed to enhance the separability of epileptic and non-epileptic events in binary classification tasks. Our study, while utilizing a more complex model, did not focus on these specific features, which may have contributed to the difference in performance. Secondly, binary classification tasks, such as those performed by Tuncer and Bolat (2022b), are generally less complex than multi-class classification tasks. Their model was optimized for binary classification, allowing it to achieve higher accuracy in those specific tasks. Our model was designed to handle a wider range of classification challenges, including more challenging three-category and five-category scenarios.

In three-category classification tasks (A-D-E, B-C-E, B-D-E, AB-CD-E), our proposed approach demonstrates the best performance. For the A-C-E classification task, our model achieves a recognition accuracy of 98.88%, slightly lower by 0.45% compared to the approach discussed in Pandey et al. (2023). Notably, the performance of the method in Pandey et al. (2023) was not assessed for the model’s generalization ability and was limited to a three-category classification problem in their literature. In the five-category classification task, the method (Shanmugam and Dharmar, 2023) achieves the highest accuracy, closely followed by our model, with an epilepsy recognition accuracy of 92.50, 1.23% higher than our proposed model. However, our model’s accuracy in the AB-CD-E classification task exceeds that of Shanmugam and Dharmar (2023) by 2.15%. Therefore, our model exhibits superior efficacy and adaptability in various epilepsy recognition and classification tasks compared to other methods.

Table 6 offers a comparative analysis of the ResBiLSTM method against recent state-of-the-art studies for classifying the same seven epileptic seizure types. Notably, ResBiLSTM outperforms other methods in terms of classification performance. Li et al. (2020) introduced a channel-embedding spectral-temporal squeeze-and-excitation network (CE-stSENet) with maximum mean discrepancy-based information maximizing loss, achieving an accuracy of 92.00% and a weighted F1 score of 93.69%. Jia et al. (2022) developed variable weight convolutional neural networks (VWCNNs) for seizure classification, attaining an accuracy of 91.71% and a weighted F1 of 94.00%. Zhang et al. (2022) implemented a variational mode decomposition (VMD) technique and nonlinear twin support vector machine (NLTWSVM), recording an accuracy of 92.29% and weighted F1 of 92.30%. Li et al. (2022) utilized fast Fourier transform (FFT) and a graph-generative neural network (GGN) for dynamic brain functional connectivity analysis, holding out (HO) 2/3 of EEG data for training and 1/3 for testing, achieving 91.00% accuracy and weighted F1. Huang et al. (2023) proposed an end-to-end 3D convolutional multiband seizure-type classification model with attention mechanisms, reaching an accuracy of 94.47% and weighted F1 of 94.38%. In comparison, our ResBiLSTM model demonstrated excellent classification performance, achieving an accuracy of 94.76% and a weighted F1 score of 94.77% with 5-fold CV, and an accuracy of 95.03% and a weighted F1 score of 95.03% with 10-fold CV.

**Table 6:** Comparison with recent state-of-the-art approaches for classifying the same seven epileptic seizure types using the TUSZ dataset.

Publication year and author	Strategy	Features	Methodology	Acc(%)	Weighted F1(%)
Li et al. (2020)	CV (5 folds)	Raw EEG	CE-stSENet	92.00	93.69
Jia et al. (2022)	CV (5 folds)	Raw EEG	VWCNNs	91.71	94.00
Zhang et al. (2022)	CV (10 folds)	VMD	NLTWSVM	92.29	92.30
Li et al. (2022)	HO (2:1)	FFT	GGN	91.00	91.00
Huang et al. (2023)	CV (5 folds)	Raw EEG	3D-CBAMNet	94.47	94.38
ResBiLSTM (Proposed)	CV (5 folds)	Raw EEG	ResBiLSTM	94.76	94.77
ResBiLSTM (Proposed)	CV (10 folds)	Raw EEG	ResBiLSTM	**95.03**	**95.03**

The bold font highlights the best result among the different methods.

The ResBiLSTM model not only demonstrates excellent performance on single-channel EEG datasets (Bonn dataset) but also excels on multi-channel EEG datasets (TUSZ). This further indicates that the ResBiLSTM model, with its well-integrated capabilities of local spatial feature extraction by the ResNet and long-term temporal dependency modeling by the BiLSTM network, possesses strong generalization ability.

## 5 Limitations and future directions

In this study, we presented the ResBiLSTM model for epileptic seizure detection, demonstrating robust performance on both the Bonn and TUSZ datasets. However, it is crucial to acknowledge the intrinsic variability and potential imprecision inherent in EEG signals, which pose significant challenges for machine learning model. The variability of EEG signals, caused by factors such as physiological differences, electrode placement inconsistencies, environmental interference, subject state changes, and equipment variability, can significantly impact model performance. While effective in controlled datasets, the current ResBiLSTM model may encounter difficulties in handling noisy and atypical data, which are common in real-world clinical settings. Two key limitations point towards areas for further research. Firstly, our current approach’s handling of data uncertainty is a limitation. The model treats the input data deterministically, potentially leading to less robust performance in the presence of substantial noise and outliers. Secondly, while our study utilizes the Bonn and TUSZ datasets, we recognize the importance of expanding our training data to enhance the model’s generalizability to unseen data. A more extensive and diverse dataset can help mitigate the risks of overfitting and improve the robustness of the model in real-world applications.

Incorporating fuzzy logic could enhance the model’s resilience to noisy and atypical data variations (Versaci et al., 2022; Zhou et al., 2022). To address the issue of EEG signal uncertainty, we plan to integrate fuzzy logic principles in our future research. Fuzzy logic can manage data imprecision and ambiguity by providing probabilistic or membership degree-based evaluations, thus reflecting the uncertainty inherent in EEG signal classification. To expand the number of training data, future research will focus on incorporating additional public EEG datasets. By including data from a variety of sources, we aim to provide our model with a broader spectrum of EEG signal characteristics. This diversity is essential for training a model capable of handling the wide range of variations present in real-world EEG data. Moreover, we will explore the generation of synthetic data through data augmentation techniques, such as employing generative adversarial networks (GANs), to enhance the model’s ability to generalize across different scenarios.

## 6 Conclusion

With the increasing volume and complexity of EEG data, DL approaches demonstrate their capability to handle the chaotic nature of EEG signals, creating new possibilities in challenging biomedical applications such as epileptic seizure detection. This work introduces a novel hybrid DL model that combines the strengths of a CNN-based network and an RNN-based network, utilizing ResNet for extracting local spatial features and BiLSTM for learning temporal dependencies. Experimental results from the Bonn and TUSZ datasets underscore the efficacy and adaptability of our proposed ResBiLSTM model across a range of epileptic seizure detection scenarios. Moreover, the proposed methodology can be utilized for identifying other related disorders, with significant implications for clinical diagnosis and analysis.

## Data availability statement

Publicly available datasets were analyzed in this study. This data can be found here: the Bonn dataset is available at https://repositori.upf.edu/handle/10230/42894, and the TUSZ dataset can be found at https://isip.piconepress.com/projects/nedc/html/tuh_eeg/#c_tusz.

## Author contributions

WZ: Writing – review & editing, Software, Methodology, Investigation, Funding acquisition, Formal analysis, Conceptualization, Writing – original draft. W-FW: Writing – review & editing, Project administration, Investigation, Conceptualization. LMP: Writing – review & editing, Investigation. B-CZ: Writing – original draft, Visualization, Validation. S-JW: Writing – review & editing, Resources, Formal analysis. S-XX: Writing – review & editing, Funding acquisition, Formal analysis, Data curation. D-ZW: Writing – review & editing, Visualization, Investigation. H-FZ: Writing – review & editing, Investigation, Formal analysis.
